# Thrombosis and myocardial infarction: the role of bioresorbable scaffolds

**DOI:** 10.20517/jca.2022.41

**Published:** 2023-01-01

**Authors:** Massoud A. Leesar, Marc D. Feldman

**Affiliations:** 1 Division of Cardiology, University of Alabama and Birmingham Veterans Affairs Medical Center, Birmingham, AL 35294, USA.; 2 Division of Cardiology, the University of Texas Health, San Antonio, TX 78229, USA.

**Keywords:** Bioresorbable scaffolds, coronary artery disease, intravascular imaging, scaffold thrombosis

## Abstract

Coronary atherosclerosis is a leading cause of death as a result of coronary thrombosis and acute myocardial infarction. Drug-eluting stents (DES) have dramatically improved the treatment of coronary artery stenosis. However, stent thrombosis (ST) and in-stent-restenosis (ISR) have remained a vexing limitation of the DES. After DES implantation, despite taking dual antiplatelet (DAPT) therapy, very late ST results in myocardial infarction and death. This occurs regardless of the type of polymer or antiproliferative agent used in the contemporary DES. Such adverse events occur at a rate of approximately 2% to 3% per year after the first year, which have been attributed to strut fractures, loss of vessel compliance, and neoatherosclerosis. Bioresorbable scaffolds (BRS) have been introduced to overcome the above shortfalls and to a “leave nothing behind” approach. While BRS are novel and interesting, the initial experience with BRS was hampered by the increased rate of thrombosis compared with DES. Accordingly, in this review, we summarized underlying mechanisms leading to BRS failure and provided insights into optimizing BRS deployment with intravascular imaging. In addition, we outlined the perspectives of new generations of BRS with thinner struts and new designs as well as alternative materials to improve outcomes.

## INTRODUCTION

Coronary atherosclerosis is the leading cause of death as a result of intracoronary thrombosis and acute myocardial infarction^[1]^. Coronary plaque rupture is implicated in the majority of coronary thrombosis; other etiologies include plaque erosion and calcified nodule^[2]^. Coronary thrombosis can also propagate atheroma formation leading to stenosis of the coronary arteries^[2]^. Patients with stenosis of coronary arteries frequently present with angina and reduced quality of life.

Percutaneous coronary intervention (PCI) with second-generation drug-eluting stents (DES) has significantly evolved and become an important treatment strategy among patients presenting with myocardial infarction or angina^[3]^. The radial strength of DES and improved deployment technique have led to optimal stent deployment in complex lesions^[3]^. However, there are still some limitations with DES, including stent thrombosis (ST) and in-stent-restenosis (ISR)^[3]^. Current guidelines^[4]^ recommend dual antiplatelet therapy (DAPT) for 12 months after DES implantation in patients with acute coronary syndromes (ACS) and 6 months for stable angina to reduce the risk of bleeding. In particular, among the elderly population with an increased risk of bleeding, the DAPT duration may be reduced to one month^[4]^. However, very late (> 12 months) DES thrombosis is a major drawback of DES because the majority of patients would stop taking P2Y_12_ inhibitors at 12 months. Such adverse events occur at a rate of approximately 2% to 3% per year after the first year of DES implantation, which have been attributed to strut fractures, loss of vessel compliance, and neoatherosclerosis^[5,6]^. Another limitation of DES includes instent restenosis after the stenting of a distal segment of a coronary artery, which could interfere with bypass surgery^[7]^.

**Figure 1.**
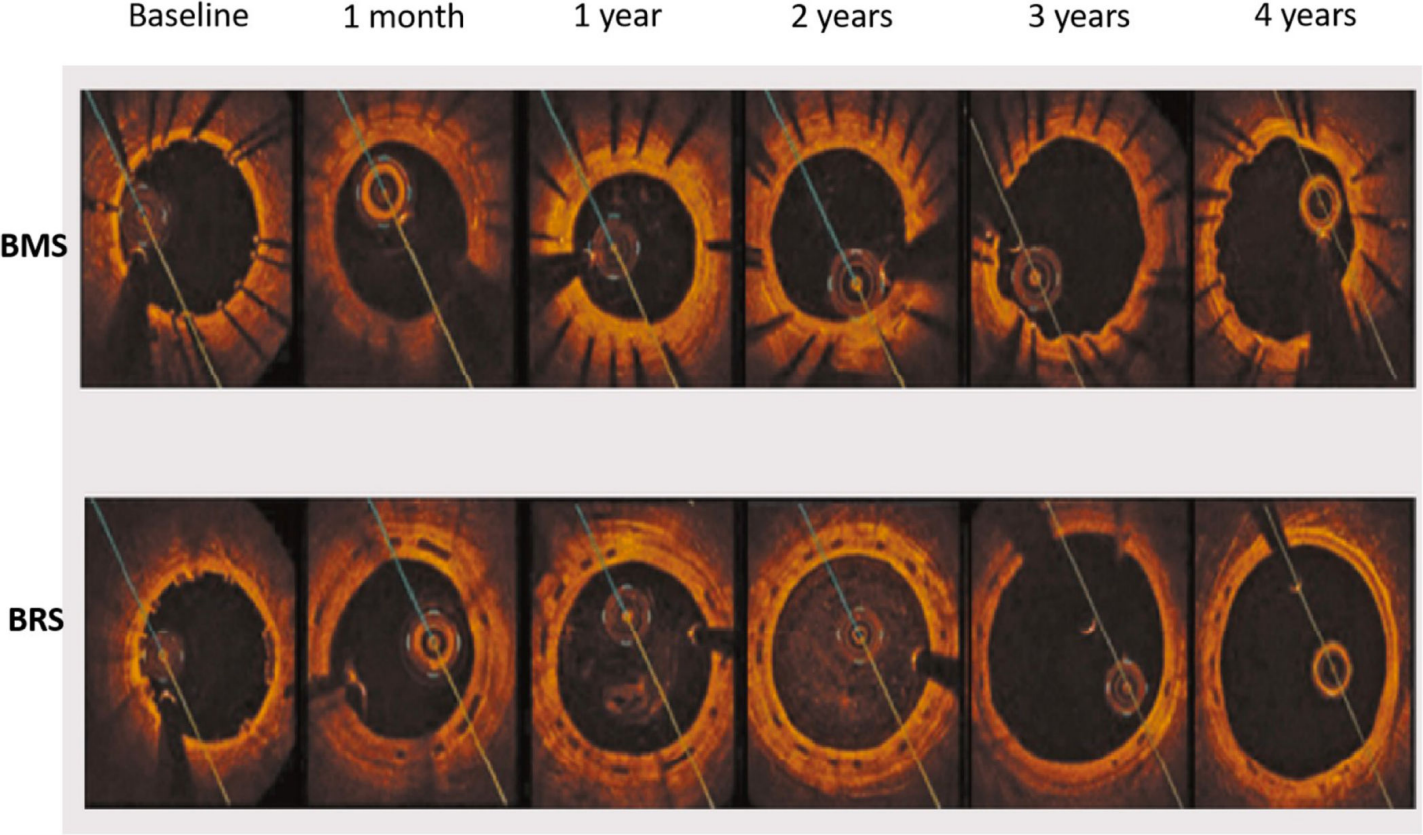
Lumen area changes by OCT after BMS (top) *vs.* BRS (bottom) deployment in swine model during 4-year period. A significant increase in lumen area of BRS *vs.* BMS occurred between 2 and 4 years, as a result of positive vessel remodeling and plaque regression. OCT: Optical coherence tomography; BMS: Bare-metal stents; BRS: Bioresorbable scaffolds.

On the other hand, bioresorbable scaffolds (BRS) have been introduced as a potential solution to avoid the above adverse events caused by permanent metallic devices. BRS provide temporary support to the vessel wall in the short term and then gradually degrade over time to restore the natural state of coronary arteries. The concept of temporary stent resulted in the development of the ABSORB BVS scaffold (Abbott Vascular, Santa Clara, CA, USA), which is a balloon-expandable poly-L-lactide (PLLA) scaffold coated with a thin bioabsorbable poly-D, L-lactide to release of everolimus, an antiproliferative drug with a similar drug delivery profile to that of the XIENCE V DES^[8]^. In a porcine model after implantation of BRS, intracoronary optical coherence tomography (OCT) and histology examination at 1 month and 2, 3, and 4 years showed that struts were discernible at 2 years by OCT and histological analysis^[9]^. At 3 and 4 years, both OCT and histology showed complete integration of the struts into the arterial wall^[9]^. Likewise, in a porcine model^[10]^, an OCT study of BRS *vs*. bare-metal stent (BMS) showed that at 4 years, BRS induced a vascular healing response comparable to BMS. However, there was expansive vascular remodeling only in the BRS group [Figure 1].

While eliminating the metallic structure, it was speculated that BRS could reduce the risk of very late acquired stent thrombosis owing to the fracture or fatigue of DES. Furthermore, by improving endothelial function, neoatherosclerosis might be prevented. Despite the above theoretical speculations, the meta-analysis of randomized trials^[11,12]^, comparing BRS with contemporary cobalt-chromium everolimus-eluting stents (EES), has shown that BRS resulted in higher adverse event rates than EES at 3 years. The failure of BRS has been attributed largely to the thick struts with suboptimal implantation technique. Subsequently, a large-scale randomized trial with 3 year-follow-up^[13]^ demonstrated some safety issues with BRS leading to its withdrawal from the market in 2017. Table 1 displays the advantages and disadvantages of DES as compared with BRS.

Accordingly, in this review, we summarized underlying mechanisms leading to BRS failure and provided insights into optimizing BRS deployment by intravascular imaging. In addition, we outlined the perspectives of new generations of BRS with tinner struts and alternative materials to improve outcomes.

### Early BRS thrombosis

**Figure 2.**
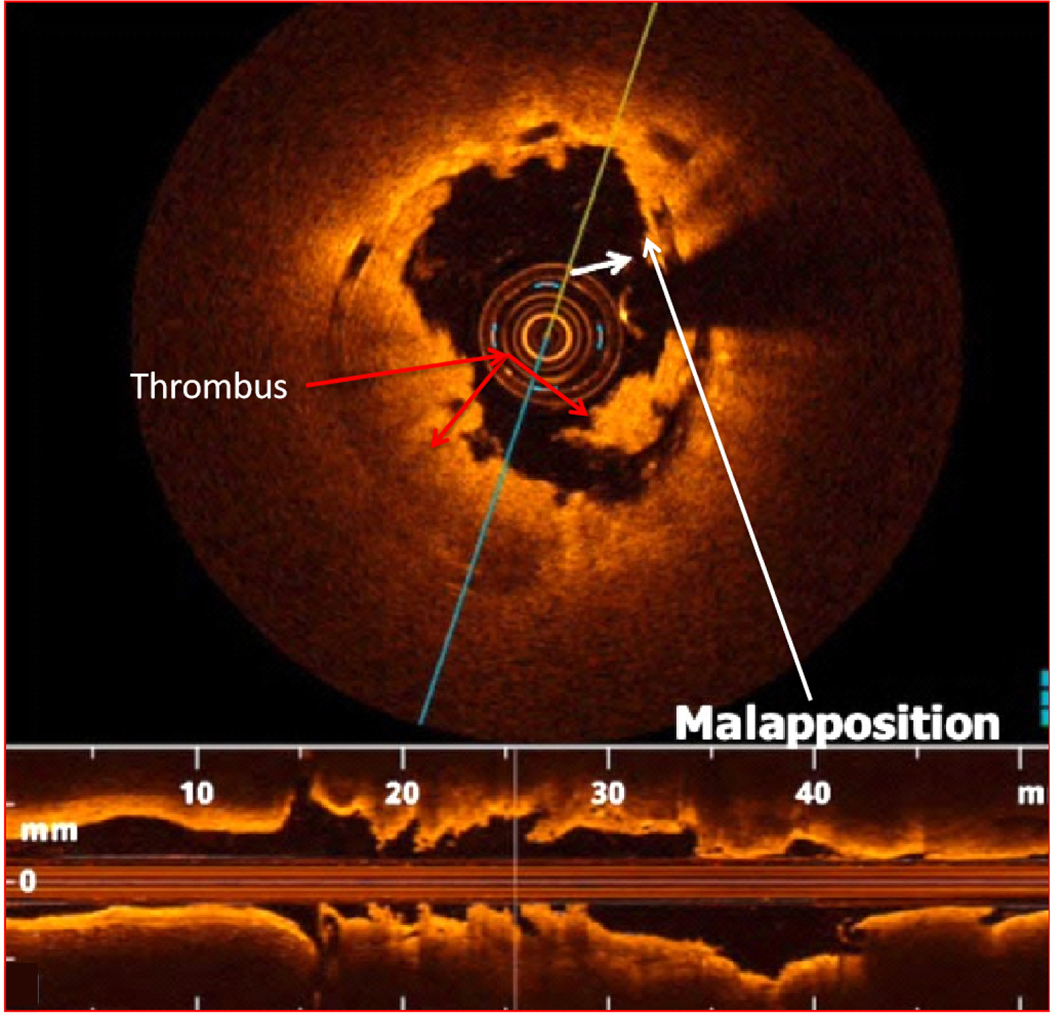
Early scaffold thrombosis after 6 days of BRS deployment. OCT shows the evidence for BRS malapposition (as shown by the white arrows and platelet thrombus in the lumen as shown by the red arrows. BRS: Bioresorbable scaffolds; OCT: optical coherence tomography.

**Figure 3.**
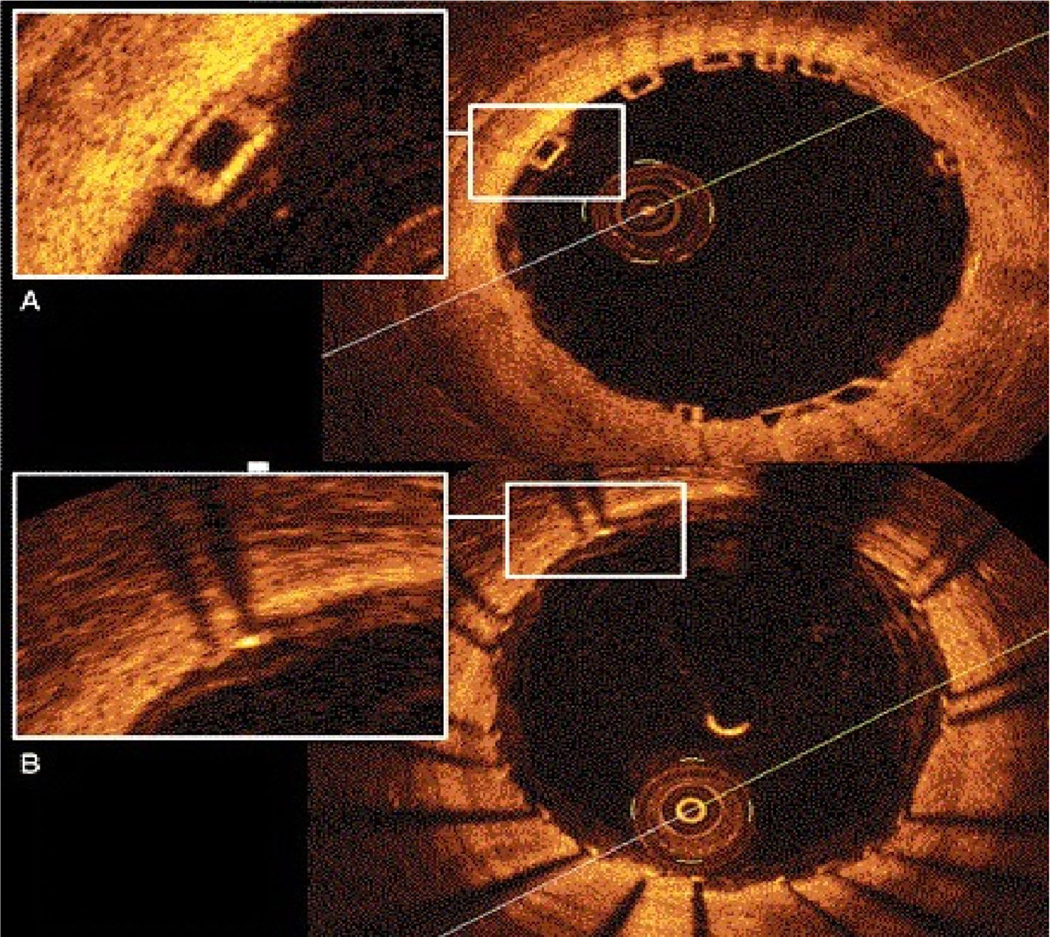
OCT imaging of the BRS *vs*. Drug-eluting stents. (A) OCT image of BRS. The struts are translucent, which leads to excellent imaging of the artery; (B) OCT images of metallic stent. The metallic struts are not translucent to the OCT and that led to the typical shadow into the vessel wall with “sunflower artifact”. OCT: Optical coherence tomography; BRS: bioresorbable scaffold.

**Figure 4.**
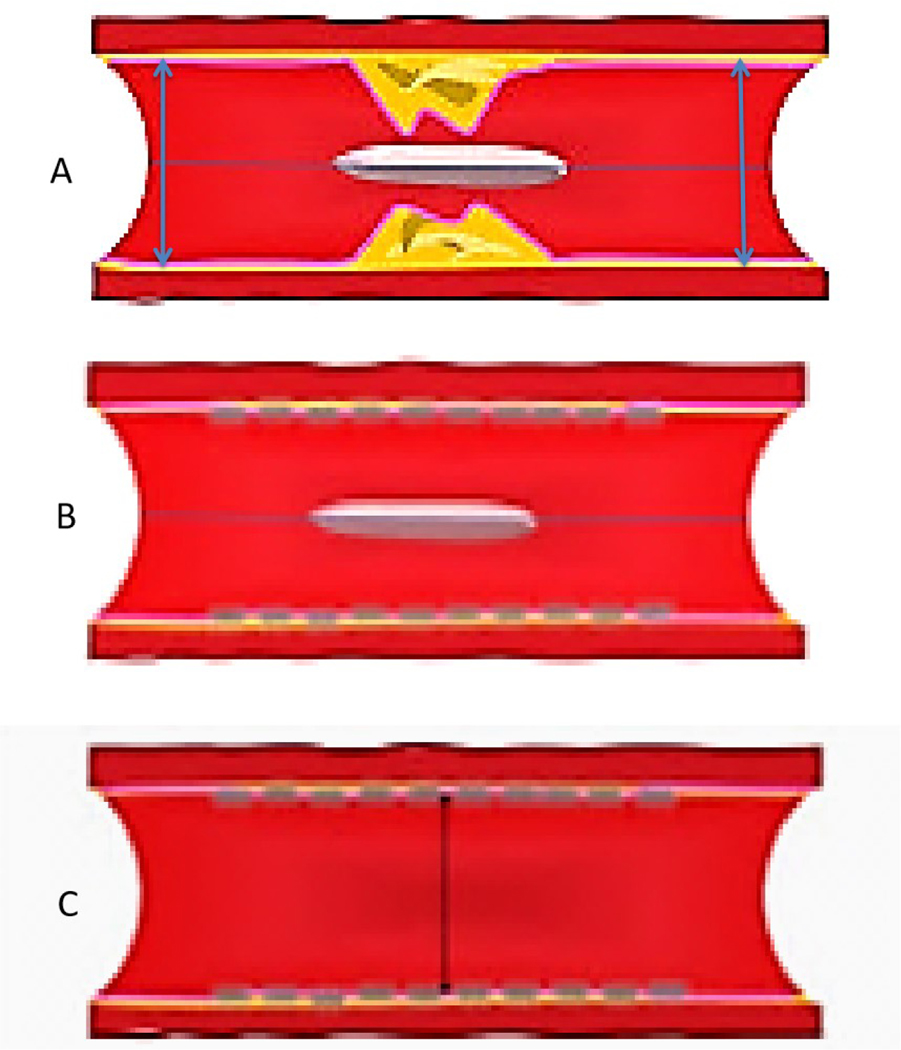
Schematic representative image of BRS deployment in the coronary artery. (A) BRS across the lesion; (B) After BRS deployment and post-dilation; and (C) final results showing struts are apposed against the vessel wall. BRS: Bioresorbable scaffold.

**Figure 5.**
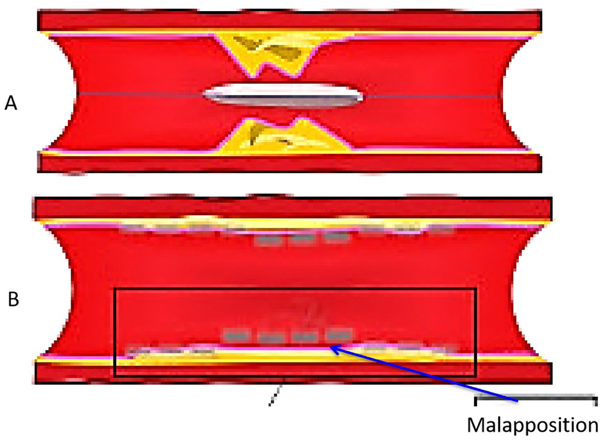
Schematic representative image of suboptimal BRS deployment in the coronary artery. (A) BRS across the stenosis; (B) After BRS deployment and post-dilation, struts are malapposed, as shown by the arrow. BRS: Bioresorbable scaffold.

Several studies reported that stent undersizing is a key factor for stent thrombosis among patients undergoing DES or BRS^[14–16]^. The timing of BRS thrombosis is defined according to the Academic Research Consortium criteria for stent thrombosis as early (< 30 days after implantation), late (> 30 days to 1 year), and very late (> 1 year). As shown in Figure 2, a BRS was implanted in a vessel. After 6 days, the patient developed acute BRS thrombosis. OCT showed that BRS was malapposed with struts not attached to the vessel wall. In addition, there is thrombosis shown as a mass attached to the vessel wall or floating within the lumen of the vessel. OCT is an intracoronary imaging device, which provides comprehensive imaging of thrombus and dissection in the vessels more accurately than IVUS. Figure 3 demonstrates images of the BRS *vs*. DES struts attached to the vessel wall. BRS struts are 150 μm thick, which is approximately two-thirds thicker than DES and that makes it difficult to expand. In addition, BRS, in contrast to DES, cannot be over-expanded. Therefore, more precise device sizing by intravascular imaging is essential. In the presence of circumferential calcification by OCT or IVUS, BRS cannot be adequately expanded and DES is preferred after performing rotational atherectomy. As shown schematically in Figure 4, BRS was sized accurately and expanded optimally with excellent results. Figure 5 shows schematically that BRS was not optimally deployed and the presence of significant malapposition.

In the Gauging Coronary Healing with Bioresorbable Scaffolding Platforms (GHOST-EU) registry^[17]^, the incidence of target lesion failure with BRS was comparable to that reported with DES^[18]^. However, the rates of definite/probable BRS thrombosis (1.5% at 30 days and 2.1% at 6 months, respectively) were higher than those reported with the second-generation DES. Furthermore, the results of ABSORB III study^[13]^ showed that stent thrombosis trended higher at 1 year (1.5% for BVS and 0.6% for the XIENCE stent). This highlights the importance of lesion selection and optimal lesion preparation before BRS implantation. In order to deploy BRS successfully and to reduce complications, an algorithm was developed called “Five P’s”: Preparation of the lesion; proper sizing of the vessel by intravascular imaging; paying attention to the expansion limits; post-dilatation using an optimally sized noncompliant balloon; and prescription for post-PCI DAPT.

### Late BRS thrombosis

**Figure 6.**
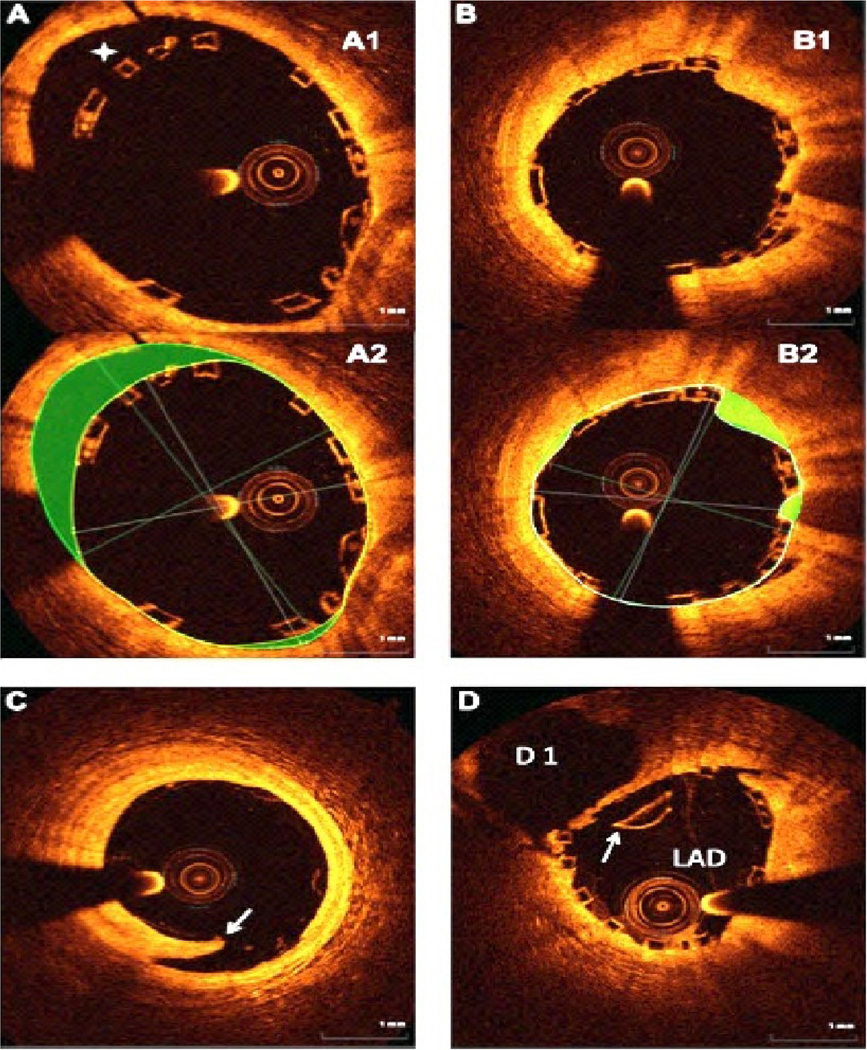
OCT analysis of BRS deployment. (A) After BRS deployment, there is incomplete strut apposition (ISA). (A1) There are 4 malapposed struts between 10 and 12 o’clock position in (A1); (A2) showing the area of malapposed struts (highlighted in green); (B1) Tissue prolapse, defined as tissue protruding between the struts. The prolapse area was measured as the difference between the struts and lumen area (highlighted in green in (B2); (C) An example of edge dissection (arrow) distal to the BRS. Because of the large lumen size and small circumferential extension of dissection, no additional BRS was deployed; (D) BRS strut fracture shown by the arrow. OCT: Optical coherence tomography; BRS: bioresorbable scaffold.

**Figure 7.**
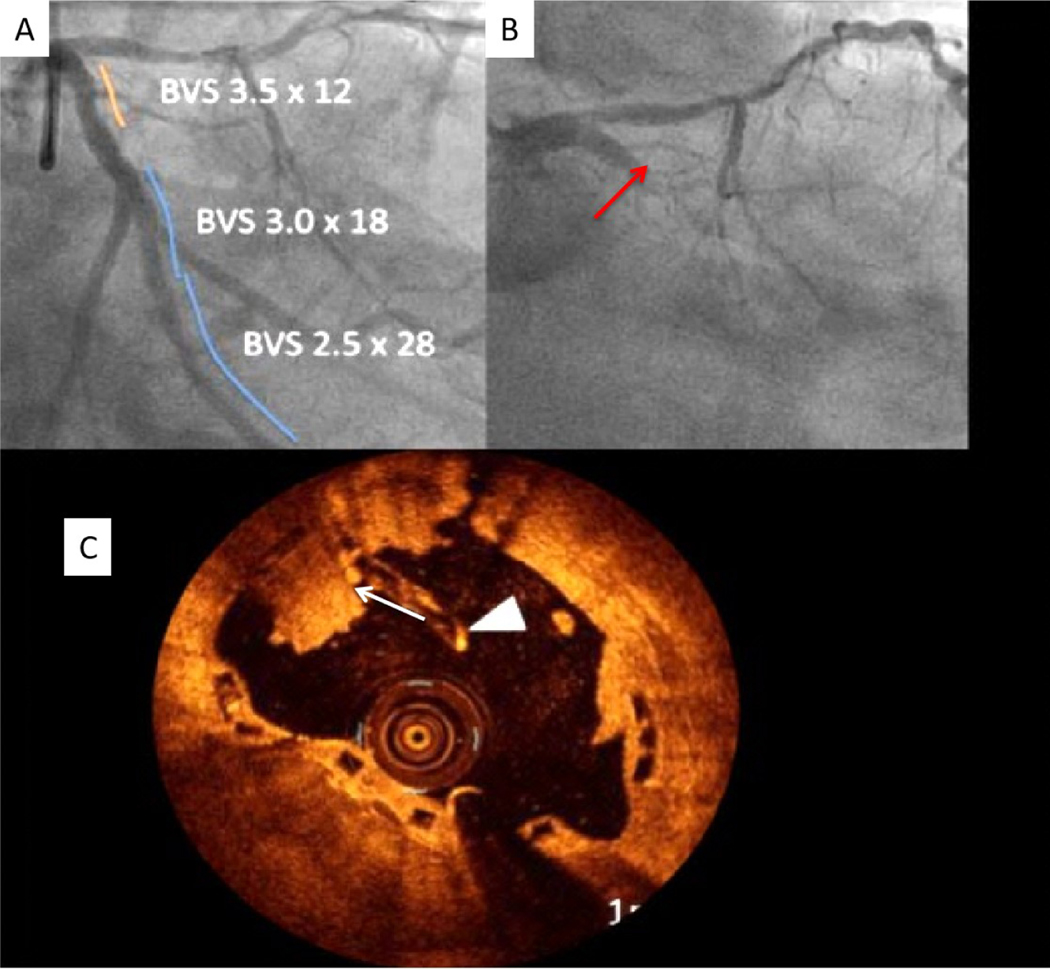
Very late BRS thrombosis. (A) Coronary angiography shows successful 3 BRSs deployment in the left circumflex coronary artery. (B) Patient was admitted with acute myocardial infarction one year after BRS deployment showing complete occlusion of the BRS. (C) OCT shows strut fracture (shown by the arrowhead) and platelet thrombus (shown by the arrow). OCT: Optical coherence tomography; BRS: bioresorbable scaffold.

Delayed healing is identified as a possible mechanism of late and/or very late stent thrombosis after implantation of the first-generation DES. Pathological studies have shown the absence of struts coverage^[18,19]^. Late-acquired stent malapposition and late drug or polymer-related hypersensitivity reactions by pathology are defined as neutrophilic or eosinophilic infiltrates^[20]^. A study^[21]^ compared vascular responses to BRS *vs*. EES in non-atherosclerotic swine and showed that the inflammation scores were greater with BRS at 6 to 36 months. OCT demonstrated a time-dependent increase in persistent low-intensity areas (PSLIA), which was associated with scaffold discontinuities^[22]^. Although the etiology of PSLIA is unknown, a BRS study in 26 coronary swine treated with EES showed good correlations between the degree of PSLIA and peri-strut inflammation at histology^[23]^. Furthermore, PSLIA has been associated with malapposition, evaginations, strut fracture, and uncovered struts as possible mechanisms of late BRS thrombosis^[23]^. Figure 6 shows several pitfalls associated with the suboptimal deployment of BRS. Figure 7 shows a case of very late BRS thrombosis in a patient who underwent successful PCI of the left circumflex (LCX) coronary artery a year ago and was admitted with acute myocardial infarction. Angiography showed 100% occlusion of the LCX artery. OCT demonstrated strut fracture associated with loss of continuity of struts, which are common causes of BRS failure.

### Randomized trials of BRS vs. DES

The results of randomized trials of BRS *vs*. DES are summarized in Table 2. ABSROB BVS, compared with contemporary EES, resulted in higher adverse event rates than EES. This has been attributed to the thicker struts and suboptimal implantation technique^[13,24–28]^. In this respect, the ABSORB III trial^[13]^ [Table 2] demonstrated that, in 2008 patients with coronary artery disease randomized to the Absorb BRS *vs*. EES, the primary composite endpoint of target lesion failure at 3 years occurred in 13.4% of the Absorb BRS patients and 10.4% of the EES patients (*P* = 0.06). In contrast, target vessel myocardial infarction at 3 years increased with BRS (8.6% *vs*. 5.9%; *P* = 0.03), as well as device thrombosis (2.3% *vs*. 0.7%; *P* = 0.01). They also showed that In the BRS group, treatment of very small vessels (those with quantitatively determined reference vessel diameter < 2.25 mm) was an independent predictor of 3-year target vessel myocardial infarction and BRS thrombosis. Recently, Kerkmeijer *et al*. reported the final results of the Amsterdam Investigator-Initiated Absorb Strategy All-Comers (AIDA) trial of the Absorb BRS *vs*. EES in 1845 patients [Table 2]^[24]^. They showed that at the 5-year follow-up period, the rate of target vessel failure was not significantly different comparing Absorb BRS *vs*. EES (17.7% *vs*. 16.1%, respectively). They showed that the increased risk of stent thrombosis with Absorb BRS continued for up to 4 years and then plateaued afterward. The rate of definite or probable stent thrombosis with Absorb BRS was not significantly different than that of EES between 4 and 5 years. As shown in Table 2, the meta-analysis^[29]^ of 6 high-quality randomized trials of 5392 patients^[13,24–28]^ demonstrated that BRS was associated with an increased risk of target vessel failure (TLF), driven by an increased risk of target vessel myocardial infarction and ischemia-driven target lesion revascularization (TLR). There was also a higher risk of definite or probable BRS thrombosis and very late BRS thrombosis compared with EES.

### Mechanisms of BRS failure

The mechanisms of BRS failure are multifactorial. Possible explanations for BRS failure are as follows: (1) limited visibility of BRS preventing the operators from performing adequate post-dilation; (2) BRS deployment in small vessels (reference vessel diameters of < 2.25 mm)^[30]^; and (3) struts fracture owing either to disruption of the plastic scaffold, malapposition, or to prolapse of struts into the lumen during dissolving process. This phenomenon is called “intraluminal scaffold dismantling”, which might be the cause of very late scaffold thrombosis^[31]^. These findings highlight the need for a meticulous operator technique, including the precise measurement of vessel dimensions by intravascular imaging, aggressive vessel preparation (predilation), and post-dilation to optimize BRS apposition. In the ABSORB III trial^[13]^, post-dilation was performed in 60% of patients after BRS deployment. In addition, intravascular imaging was performed in only 11% of patients to optimize BRS deployment. This has contributed significantly to the failure of the trial.

### Coronary imaging to improve implantation of BRS

Several studies reported the advantages of coronary imaging with OCT or IVUS to guide optimal PCI^[14–16,32,33]^. These advanced imaging modalities lead to more accurate determination of the vessel diameter and lesion length, and stent expansion. In addition, they immediately help diagnose edge dissections. The use of these devices would reduce the risk of BRS thrombosis and restenosis. In particular, intravascular imaging is helpful in vessel sizing before stenting. It has been shown by IVUS that stent under-expansion and malapposition would result in a higher rate of restenosis and stent thrombosis. Given the unique mechanical properties of BRS and the procedural details for successful implantation, IVUS as an adjunct to angiography has significantly reduced the rate of target vessel revascularization^[34,35]^.

As shown in Figure 6, OCT is a light-based high-resolution intracoronary imaging modality, which allows for more accurate detection and quantification of BRS malapposition, under-expansion, tissue prolapse, and stent edge dissection^[36]^. The four sides of the BRS struts can be seen by OCT without the typical shadowing observed in metallic struts [Figure 3]. OCT provides higher resolution in the range of 10–15 μm, rendering it a distinct advantage over IVUS^[37]^. The CLI-OPCI study^[38]^ showed that in 670 patients randomized to OCT *vs*. angiography groups, OCT detected complications requiring further interventions in 34.7%. In addition, the OCT group had a significantly lower risk of cardiac death, myocardial infarction, or repeat revascularization as compared with angiography at 1 year. It is worth noting that overexpansion of BRS is limited as compared with modern DES. It is possible to dilate a modern DES stent 1.0–1.5 mm over the specified diameter. However, BRS can only be dilated 0.5 mm over the specified diameter. Exceeding these limits would increase the risk of ring fractures with subsequent strut protrusion into the lumen. This can trigger late complications such as stent thrombosis or target vessel restenosis. Furthermore, after BRS deployment, OCT can identify inadequate BRS expansion or incomplete struts apposition, which could lead to stent thrombosis or restenosis.

### The use of OCT for follow-up BRS implantation

The use of DES has significantly inhibited endothelialization and minimized ISR. Given that DES are permanent and may be associated with durable risk, including restenosis and/or thrombosis as a result of persistent uncovered struts, stent fracture, and/or neoatherosclerosis^[3]^. On the other hand, BRS might be advantageous to DES because of complete resorption. After BRS implantation, the optimal duration of dual antiplatelet therapy (DAPT) has yet to be determined. There is no data with BRS indicating that the duration of DAPT can be shortened to 3 months. In this respect, follow-up of BRS after deployment by OCT may be required to determine the percentages of covered struts, the extent of neointima coverage, and struts dismantling. These assessments provide knowledge on the long-term performance of BRS and its role in modern PCI. Finally, OCT is a key imaging technique for the accurate assessment of scaffold absorption during follow-up. In the pilot ABSORB cohort (A) study^[39]^, OCT revealed that 100% of the scaffold struts were fully covered by tissue and apposed. After 2 years, struts were resorbed with complete integration of the scaffold into the vessel wall. Furthermore, serial OCT studies showed that after BRS implantation, as compared with bare-metal stents, the vessel lumen enlarged and the plaque/media diminished [Figure 1].

### The use of IVUS for stent optimization

IVUS is well known for detecting suboptimal stent results and improving the outcome of PCI with no safety concerns. The 5-year follow-up of the IVUS-XPL (Impact of Intravascular Ultrasound Guidance on the Outcomes of Xience Prime Stents in Long Lesions) randomized trial^[34]^ showed that the use of IVUS, compared with angiography, significantly improved outcomes. Along the same line, the ULTIMATE (Intravascular Ultrasound Guided Drug-Eluting Stents Implantation in All-Comers Coronary Lesions) trial^[35]^ showed that the use of IVUS during PCI resulted in significantly lower rates of target vessel failure and ST at the 3-year follow-up. An updated meta-analysis^[40]^ of 10 randomized trials of 5060 patients of IVUS-*vs*. angiography-guided PCI demonstrated that IVUS-guided PCI led to significantly lower adverse events at 14-month follow-up. Taken together, the recent randomized trials^[34,35]^ and the above-updated meta-analysis^[40]^ demonstrate that the use of IVUS, compared with angiography-guided PCI, significantly improved outcomes.

**Figure 8.**
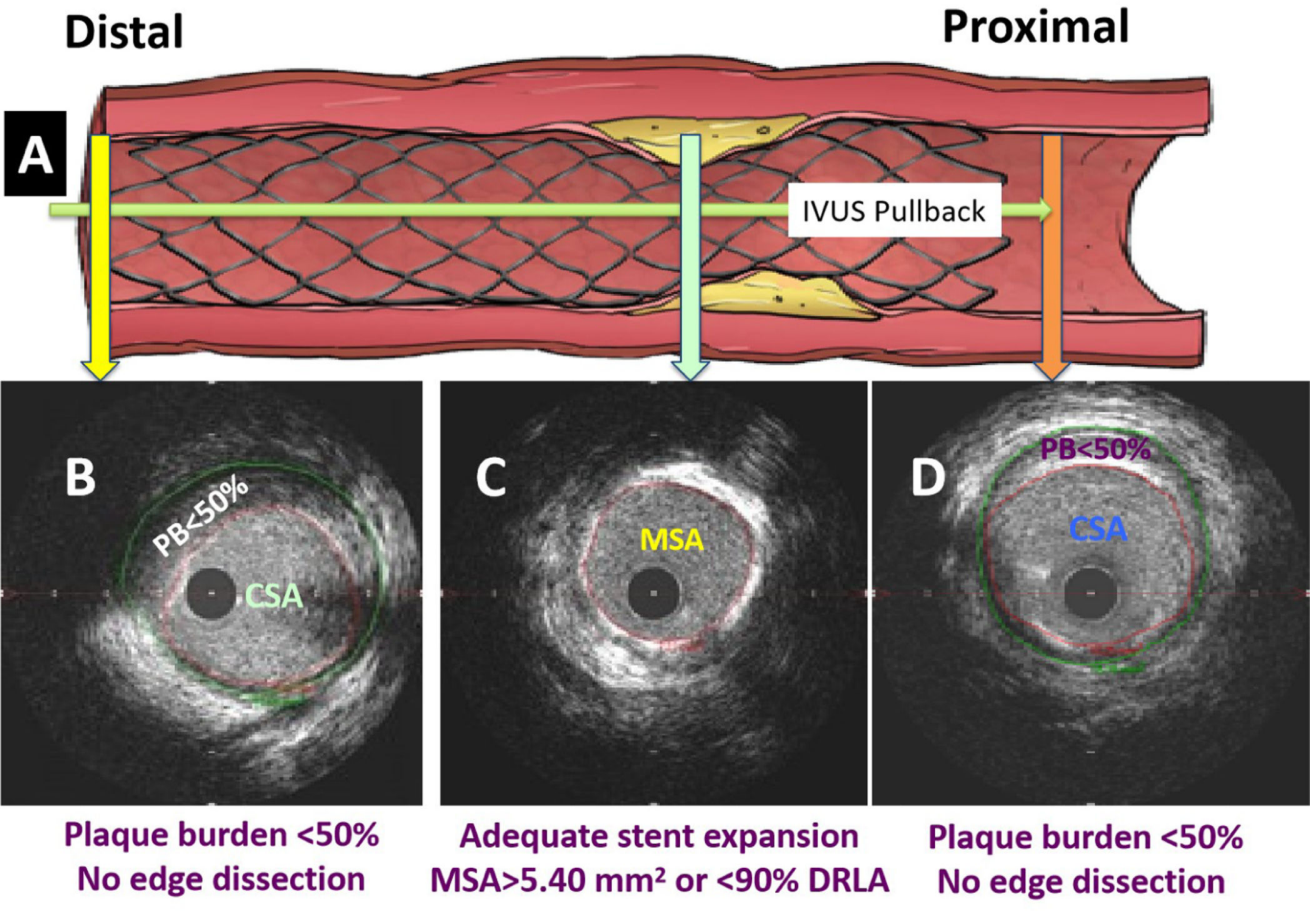
The use of a standard approach shown schematically by IVUS. (A) Following stent deployment and post-dilation, IVUS was performed using the automatic pullback recordings from distal part of the vessel to proximal; (B and D) Plaque burden (PB) was measured in a segment at the 5 mm proximal or distal to the stent edges. If the PB was > 50% or in the event of edge dissection, a stent was deployed; (C) at the lesion site, if minimum stent area (MSA) was < 5.4 mm^2^ or < 90% of distal reference lumen area (DRLA), further post-dilation of the stent was performed using high-pressure balloon inflation sized to the distal EEM diameter. Repeat IVUS pullback was performed to investigate whether the optimal stent results, defined as MSA ≥ 5.4 mm^2^ or ≥ 90% of DRLA at the lesion site and plaque burden < 50% at the stent edges with no edge dissection, were achieved.

As shown in Figure 8, after post-dilation using a noncompliant balloon sized to the distal reference external elastic membrane (EEM) diameter, repeat IVUS showed that the minimum stent area (MSA) was > 5.4 mm^2^ or > 90% of the distal reference lumen area [DRLA]). Likewise, the plaque burden (PB) was < 50% at the stent edges. We showed that these standard criteria for optimal stent expansion improved the stent results in > 85% of patients^[41]^. It has been shown (42) by OCT that the sizing of stent to the EEM diameter led to an improvement of stent expansion safely with no dissection or perforation^[42]^.

Recently, Costantini *et al*. reported the outcome of BRS implantation guided by IVUS^[43]^. In this study, the authors implanted BRS in 171 lesions (141 vessels). In 31% of patients, an additional intervention was required. At follow-up, there was no stent thrombosis, myocardial infarction or death. The rate of target lesion failure was 4%. While the above study is encouraging, the excellent outcome of the aforementioned study indicates that IVUS optimization is required to improve outcomes.

## FUTURE PERSPECTIVES OF BRS

The majority of BRS scaffolds are made of lactate polymers. BRS is categorized as polymeric resorbable scaffolds or metallic resorbable scaffolds (MRS). Other materials include magnesium alloys, tyrosine copolymers, and iron. Table 3 displays the design of the current contemporary BRS. Notably, none of the above contemporary BRS is approved by FDA. Table 4 shows the outcomes of contemporary BRS in non-randomized trials.

It is worth noting that strut thickness is one of the shortfalls of BRS, leading to stent thrombosis^[44]^. The use of thicker struts (greater than 150 μm) in a small vessel without sizing by imaging or lack of post-dilation might lead to stent thrombosis. Other issues, including polymer and scaffold disintegration, have also been associated with stent thrombosis^[31]^. In this respect, new designs of BRS with reduced strut thickness can help improve crossing profile and deliverability. Furthermore, the advent of thinner struts could reduce shear stress and thrombosis, as a result of improving endothelization^[45]^. Thinner struts have also been shown to reduce restenosis and periprocedural myocardial infarction rates^[46]^. As shown in Table 4, BIOSOLVE-IV-registry^[47]^ investigated the safety and performance of the Magmaris sirolimus-eluting bioresorbable magnesium scaffold in 1075 patients [Tables 1 and 2]. They showed that the Kaplan-Meier estimate of TLF at 12 months was 4.3%, including 3.9% target lesion revascularizations, 0.2% cardiac death, and 1.1% target-vessel myocardial infarction. Definite/probable scaffold thrombosis occurred in five patients (0.5%), which is comparable to DES. However, randomized trials are needed to investigate the outcomes of the above device with EES.

Given that the rate of target lesion revascularization (TLR) of 0.2% per year with the second-generation DES^[48]^, BRS would need to perform as equally as DES in the short term and better in the long term. However, this was not achieved with the first-generation BRS and continuous iterations of contemporary BRS are needed. In this respect, randomized trials of contemporary BRS *vs*. EES are needed to assess the non-inferiority of the contemporary BRS *vs*. EES. In particular, the expanded use of intravascular imaging could lead to optimal deployment of BRS and that might significantly improve the outcome. In addition, future trials are in progress to investigate the timeline of resorption with the current BRS. Given that the thickness of the contemporary BRA has significantly reduced, the process of resorption could be faster and thus the duration of DAPT might be shortened. In addition, the outcomes of polymeric resorbable scaffolds as compared with metallic resorbable scaffolds (MRS) are unknown.

### Procedural considerations

Given significant advancement in the design of BRS, it is essential that BRS be deployed optimally. Notably, BRS under-expansion and malapposition were the common hurdles leading to adverse events in the past^[49]^. The use of the PSP technique has resulted in reducing the incidence of BRS thrombosis in a registry study^[50]^. Given that randomized trials of contemporary BRS guided by intravascular imaging are needed to determine their efficacy and safety as compared with EES.

### Dual antiplatelet therapy after BRS

While the time curve of BRS resorption is a determining factor for prevention of BRS thrombosis, DAPT should be continued until scaffold resorbs completely. However, this would need to be assessed considering the increased bleeding risk with DAPT. The new design of the scaffold with shorter time to resorption could reduce DAPT duration and that might become more suitable in patients with an increased risk of bleeding. Current European guidelines recommend a minimum of 12 months of DAPT for polymeric and metallic resorbable scaffolds (class IIA C)^[4]^.

## CONCLUSIONS

The BRS technology still holds promise. Notably, opportunities were missed and lessons were learned from the ABSORB program. The emerging data supports the potential clinical benefits of contemporary BRS technology. In this respect, the use of routine intravascular imaging for optimal BRS deployment and the use of thinner struts with contemporary BRS technology might pave the way to improving outcomes. Taken together, thinner struts, newer design characteristics, appropriate patient selection, and standardized techniques of implantation guided by intravascular imaging might lead to better outcomes and improve the care of patients.

## Figures and Tables

**Table 1.** Advantages and disadvantages of DES compared with BRS

	DES (advantages)	DES (disadvantages)	BRS (advantages)	BRS (disadvantages)

Stent thrombosis	X			X
Strut thickness	X			X
Struts dismantling	X			X
Late and very late stent thrombosis		X	*Possible	
Very late struts fracture		X	*Possible	
Neoatherosclerosis		X	*Possible	
Deployment technique	X			X
Malapposition	X			X
Underdeployment	X			X
Shear stress	X			X
Restenosis	X			X
Duration of DAPT	X			X
Delivery profile	X			X

* Pending future trials with contemporary BRS. DES: Drug-eluting stents; BRS: bioresorbable scaffolds.

**Table 2.** Randomized trials of ABSORB BVS *vs.* everolimus-eluting metallic stents

Trials	AIDA	ABSORB III	ABSORB II	ABSORB Japan	ABSROB China	EVERBIO II

**Year published**	2017	2017	2016	2016	2016	2015
**Randomized**	**BRS/DES**	**BRS/DES**	**BRS/DES**	**BRS/DES**	**BRS/DES**	**BRS/DES**
**Patients randomized, n**	924/921	1322/686	335/166	266/134	241/239	78/80
Age, *y*, mean	64/64	64/64	62/61	67/67	57/58	65/65
Male, %	73/76	71/70	76/80	79/74	72/73	80/80
DM, %	19/17	32/33	24/24	36/36	25/23	22/16
Prior MI, %	19/18	22/22	28/29	16/24	17/16	14/18
ACS, %*	54/55	27/25	20/22	10/16	65/64	25/26
**Lesions**						
Grade B2-C, %	55/51	46/49	75/72	76/76	69/73	30/35
LAD, %	42/44	45/46	55/52	46/42	45/42	46/39
LCX, %	42/26	29/23	20/24	23/26	26/31	25/19
RCA, %	32/29	26/31	25/23	31/31	29/27	25/36
Post-stent dilation, %	74/49	61/59	63/54	82/77	65/51	34/31
Follow-up, months	23	25	36	24	24	24
*TLF (n)	BRS/DES	BRS/DES	BRS/DES	BRS/DES	BRS/DES	BRS/DES
RR (95% CI)	91/78	143/53	34/8	52/16	10/11	16/13
Overall: 1.32 (1.11,1.58)	1.16 (0.87, 1.55)	1.40 (1.04, 1.89)	2.1 (1.0, 4.45)	1.64 (0.97, 2.7)	0.90 (0.4, 2.1)	1.2 (0.65, 2.4)

* Death, target vessel myocardial infarction, and ischemia-driven target vessel revascularization.

**Table 3.** The design of the current contemporary bioresorbable scaffolds

Device	Antiproliferative drugs	Back bone	Thickness of struts (μm)	Bioresorption time (months)	CE mark (yes-active)	FDA approval

DESolve (Elixir Medical)	Novalimus	PLLA	150	24–36	Yes, 2014	No
ART pure (Arterial Remodelling Technologies)	Drug-free	PDLLA	170	12–24	Yes, 2015	No
MeRes 100 (Meril Life Sciences)	Sirolimus	PLLA	180	24	Yes, 2019	No
FORTITUDE (Amaranth Medical)	Sirolimus	PLLA	150	12–24	No	No
APTITUDE (Amaranth Medical)	Sirolimus	PLLA	115	12–24	No	No
MAGNITUDE (Amaranth Medical)	Sirolimus	PLLA	98	12–24	No	No
DEFIANCE (Amaranth Medical)	Sirolimus	PLLA	85	12–24	No	No
Mirage (Manli)	Sirolimus	PLLA	125–150	14	No	No
NeoVas (Lepu Medical Technology)	Sirolimus	PLLA	180	36	No	No
Firesorb (Shanghai MicroPort)	Sirolimus	PLLA	100–125	36	No	No
Falcon (Abbott)	Everolimus	PLLA	< 100		No	No
Fantom (REVA Medical)	Sirolimus	DAT	125	12	Yes, 2017	No
Magmaris (Biotronik)	Sirolimus	Magnesium	120–150	12	Yes, 2016	No
IBS (Lifetech Scientific)	Sirolimus	Iron	70	12	No	No

CE Mark indicates the trial is active.

**Table 4.** The outcomes of contemporary bioresorbable scaffolds used in recent clinical trials

Device	Patients (*n*)	Follow-up by angiography (months)	Late lumen loss (mm)	Follow-up clinical (months)	TLF (%)	Scaffold thrombosis (%)	Ischemic-driven TLR (%)

DESolve Nx (Elixir Medical)	122	6	0.20 ± 0.32	60	7.4	0	4.1
ART Pure (Arterial Remodelling Technologies)	30	6	-	-	-	-	-
MeRes 100 (Meril Life Sciences)	108	6	0.15 ± 0.23	12	-	0	0.9
FORTITUDE (Amaranth Medical)	63	24	0.27 ± 0.37	24	4.9	1.8	5.3
APTITUDE (Amaranth Medical)	60	9	0.33 ± 0.36	24	3.4	0	0
MAGNITUDE (Amaranth Medical)	70	9	0.19 ± 0.16	9	2.9	0	0
*Mirage (Manli)	35	12	0.37 ± 0.14	12	17.2	3.4	17.2
NeoVas (Lepu Medical )	1103	-	-	12	3.0	0.5	1.7
Fantom (REVA Medical)	117	6	0.25 ± 0.40	24	4.2	0.8	2.9
Magmaris (Biotronik)	1075	12	0.52 ± 0.39	36	4.3	0.5	2.4

* Microfiber technology: Higher target lesion failure (TLF) and ischemia-driven target lesion revascularization (TLR) is probably related to a suboptimal deployment technique.

## Data Availability

Not applicable.
